# A Compact UWB Monopole Antenna with Triple Band Notches

**DOI:** 10.3390/mi14030518

**Published:** 2023-02-23

**Authors:** Han Lin, Zhongyuan Lu, Zhonggen Wang, Weidong Mu

**Affiliations:** School of Electrical and Information Engineering, Anhui University of Science and Technology, Huainan 232001, China

**Keywords:** multi-notch band, UWB antenna, monopole

## Abstract

This article presents an ultra-wideband (UWB) monopole antenna with triple band notch characteristics. The proposed antenna consists of an octagonal patch, fed with a 50 Ω line, which occupies a compact size of 40 mm × 29 mm (0.36λ × 0.26λ, λ is computed using 2.7 GHz frequency) and resonances at a relatively low frequency (2.94 GHz). Specifically, an L-shaped stub, an inverted C-shaped slot, and a pair of U-shaped resonating structures are introduced into the design, which allow antenna to generate three band notches at 3.22–3.83 GHz, 4.49–5.05 GHz and 7.49–8.02 GHz, corresponding to WiMAX band, Indian national satellite (INSAT) band, and X-band satellite frequencies, respectively. In the center of the notched band, the antenna has lower efficiency and gain, essentially indicating that the antenna has good interference rejection performance. To evaluate its performance, the proposed antenna has been fabricated and measured, and the relevant functional parameters, such as S-parameters, voltage standing wave ratio (VSWR) and radiation property, have been studied.

## 1. Introduction

Ultra-wideband (UWB) technology has great potential for application in wireless communication because of its extremely low transmission power and high data rate [1]. In recent years, research into UWB applications has gained significant attention, mainly for communication, radar and precise positioning [2,3]. Within the defined UWB range, a variety of narrowband communication systems, including the worldwide interoperability for microwave access (WiMAX) band which operates at 3.3~3.7 GHz, the Indian national satellite (INSAT) band which operates at 4.5~4.9 GHz and the X satellite communication band which operates at 7.1~8 GHz, have the potential limitation of bringing electromagnetic (EM) interference that affects the performance of UWB antenna. Therefore, an antenna with multiband filtering is required to suppress the interfering bands.

Over the years, researchers have proposed various methods to design the band-notched UWB antennas [4,5,6,7,8,9,10,11,12]. Among them, one such method is to add parasitic elements [13], stubs [14], and resonating structures on or near the radiator [15,16]. Meanwhile, another approach is to etch differently shaped slots in the radiating element or the ground plane [17,18,19,20]. The methods mentioned above can be employed to suppress single or multi-band phenomena, and the selectivity of the rejection bands depends on the effectiveness of the incorporated techniques. In [13], a parasitic strip was designed as a filter to eliminate the band limited by IEEE 802.11a and HIPERLAN/2. Progressively, four types of band-notched antennas were proposed in [14], where the first antenna connected two strips horizontally and symmetrically to the feed line, to create a single-notch band. Whereas for the second one, two quarter-wavelength open-ended slots were embedded in the feed line, and this antenna possessed a single-notch band covering the upper wireless local area network (WLAN) band. Next, the dual-band-notched characteristics were achieved by inserting the additional stubs into the rectangular slot of the second antenna. Compared with the second antenna, this third one had an additional notch band covering the lower WLAN band. Then, in the final design, the designs of the first three steps were combined to achieve three notch bands. In [15], the complementary split-ring resonators (SRRs) were etched onto the backside of the feed line in order to produce a notch that covered the satellite downlink band. In addition, the 5G and WLAN bands were notched by using a pair of electromagnetic band gap (EBG) structures. Similarly, in [16], the complementary SRRs consisted of a pair of metallic rings which were arranged close to the feeding strip in order to achieve a single-notched band. In [17], three U-shaped slots were etched onto the radiating element to obtain three notched band characteristics, and a split-ring resonator was also introduced to create an additional notch band. In [18], a meandering line slot was added in the middle of the patch to realize the triple-notch capabilities and multiband operation. In [19], the WLAN and X-band communication systems were rejected by a pair of vertical slots and a horizontal rectangular slot etched onto the same radiation patch. In [21], two identical meander line slots were etched onto the two decoupling T-shaped stubs symmetrically to achieve a notch band from 5.09 to 5.8 GHz. In [22], by introducing two different dimensions of U-shaped parasitic strips on both sides of each feed lines, frequency bands of 7.37–7.8 GHz and 9.15–9.7 GHz were successfully suppressed. More than this, two different dimensions of U-shaped slots were etched on each radiator, leading to the rejection of the lower WLAN band and the frequency range from 6.1 to 6.53 GHz. In [23], a frequency-agile band-notch function was realized for frequencies below 5 GHz by placing a single varactor diode across the gap on the rectangular strip.

The following are the novel discoveries and contributions of this work:

1. The controllable triple-notch frequencies are achieved at the WiMAX, INSAT and X-band satellite frequency bands.

2. The proposed antenna integrates multiple forms of notch structures with different shapes and techniques.

In this work, a planar UWB monopole antenna is designed, fabricated and tested. The dimensions of the stub and slot are varied to achieve the desired stopband center frequency. Furthermore, three different band-notched designs are provided for illustration, along with a description of the design concept. Lastly, the details of antenna measurement and simulation results are presented, which demonstrate a successful band-rejection capability for all three proposed band-notched designs. Above all, the proposed antenna can be a potential option for specific devices operating in WiFi 6E band.

## 2. Antenna Configuration

### 2.1. Antenna Model

**Figure 1:**
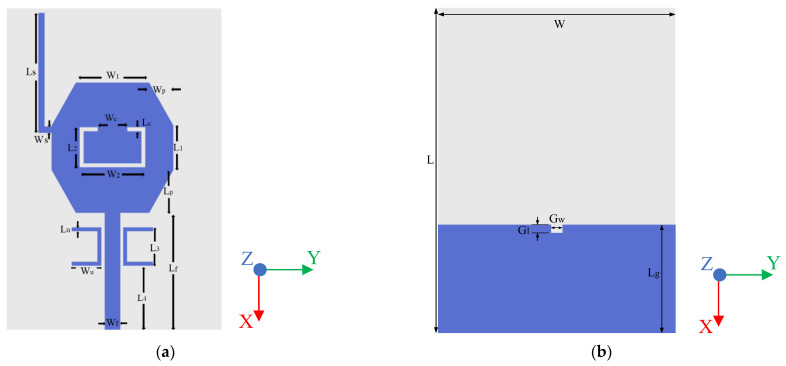
Proposed antenna structure: (**a**) Front view, (**b**) Back view.

**Figure 2:**
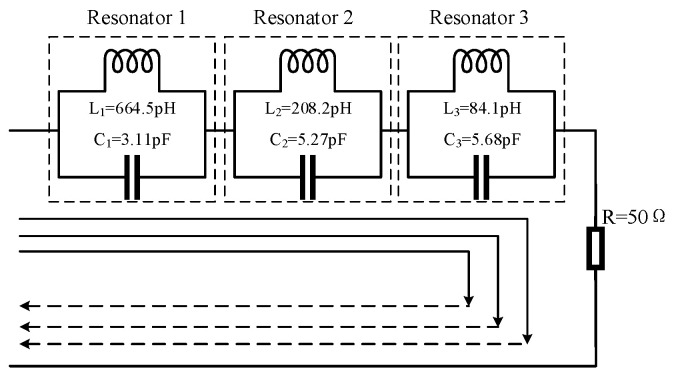
Equivalent circuit of triple-notch UWB antenna.

**Figure 3:**
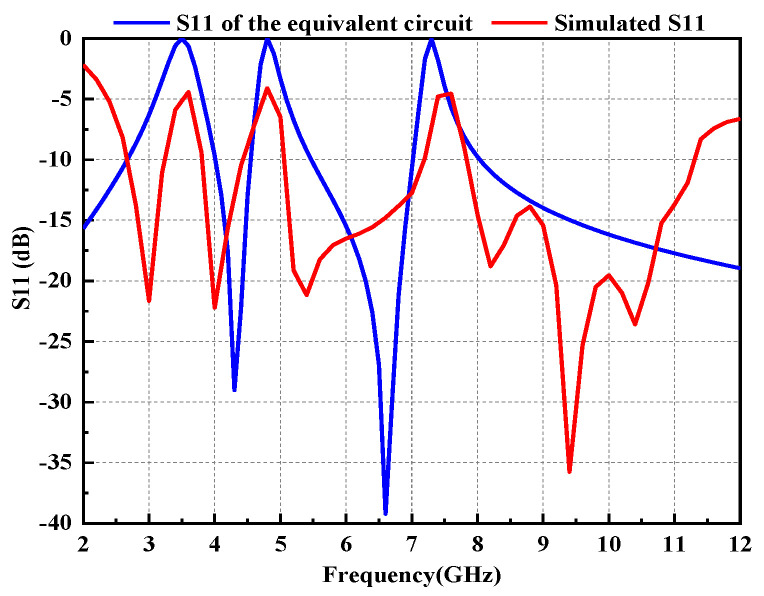
Comparison between S11 of equivalent circuit and simulated S11.

**Figure 4:**
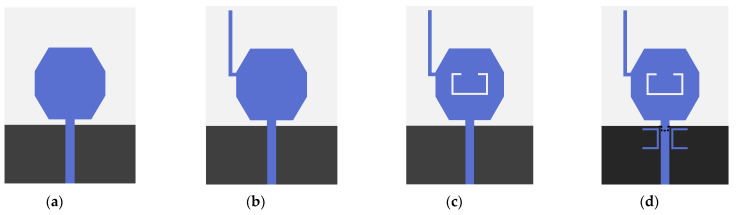
Evolution of the design process of single element: (**a**) Step-1, (**b**) Step-2, (**c**) Step-3, (**d**) Step-4.

**Figure 5:**
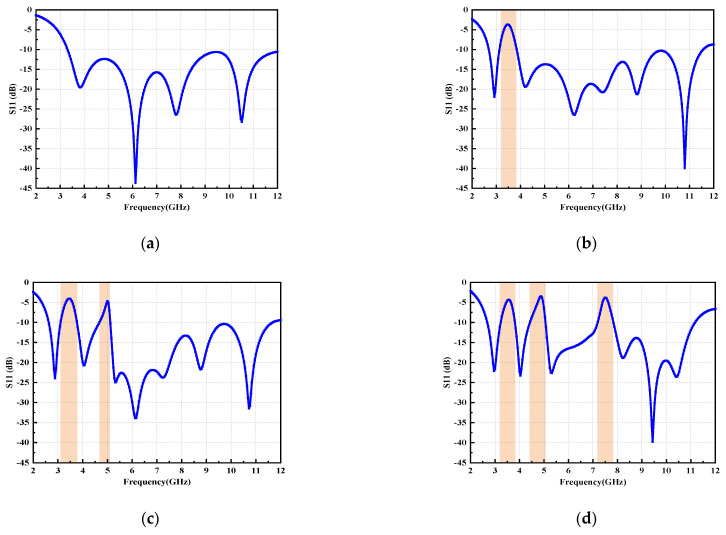
Comparison of the return loss of the four steps of the proposed antenna: (**a**) Step-1, (**b**) Step-2, (**c**) Step-3, (**d**) Step-4.

The proposed antenna is fabricated on a common and low-cost FR4 substrate (ε_r_ = 4.4) with a thickness of 1.6 mm. Moreover, a 50 Ω microstrip-line was fed by an SMA connector. The geometry and configuration of the proposed antenna have been designed and optimized using HFSS 18.0, as shown in Figure 1. Equivalent circuit of triple-notch UWB antenna is displayed in Figure 2. S11 of equivalent circuit is shown in Figure 3. Next, the design evolution process is illustrated in Figure 4, and the S-parameters for each stage of the evolution process are provided in Figure 5. Additionally, the optimized values of all the designed parameters are listed in Table 1.

Figure 2 shows the equivalent circuit of the proposed antenna, where resonator 1, resonator 2, and resonator 3 are equivalent to the L-shaped stub, inverted C-shaped slot and symmetrical U-shaped patches, which are equivalent to three LC parallel resonant circuit in the circuit. Since the circuit is open, the antenna cannot receive the signal properly, thus effectively avoiding interference from these three narrowband communication systems. The approximate value of *L* and *C* can be computed by the following formulas [24]:

(1) $$Q_{i}=\frac{f_{i}}{BW_{i}}$$

(2) $$C_{i}=\frac{Q_{i}}{2\pi f_{i}R_{i}}$$

(3) $$L_{i}=\frac{C_{i}}{{\left(2\pi f_{i}\right)}^{2}}$$

where, *i* is the number of resonator, *Q* is quality factor, *BW* is the bandwidth of each notch band, *C* is the shunt capacitance per unit in F, *L* is the shunt inductance per unit in H, *f* is the center notch frequency of notch band, and *R* is the real part of impedance at resonance frequency.

### 2.2. Design Evolution Stages of the Antenna

The details of all the stages of the design evolution process are given below:

Step-1 includes an octagonal radiation element with a rectangular ground structure (Figure 4a). This antenna operates in the UWB region, with a bandwidth of 8.75 GHz.

Step-2 introduces an L-shaped stub at the upper left corner of the octagonal patch (Figure 4b). The distance between the lower edge of the L-shaped stub and the lower edge of the substrate can be calculated as: d = L_f_ + L_p_ + L_1_ − W_s_. Here, L-shaped stub acts as an open-circuit transmission line that shorts the antenna at the relevant frequency. Thus, the effective transmission path of current is changed [25], causing the antenna to resonate at a lower frequency (2.94 GHz) and creating a notch band that is immune to WiMAX system interference.

Based on the design of step-2, an inverted C-shaped slot is etched onto the center of the octagonal patch, which defines step-3 (Figure 4c). At certain frequencies, the current path of the signal can be cut off, leading to an additional notch band that can shield from the INSAT band and has a small effect on other resonance bands [26].

The specifics of the rejection bands for the design of each stage have been listed in Table 2.

An approximate size of the notch structure can be assumed as [27]:

(4) $$L=\frac{c}{2f\sqrt{\varepsilon_{eff}}}$$

where, *c* is the speed of light, *f* is the notched center frequency, *ε_eff_* is the effective dielectric constant.

Next, a transverse U-shaped resonator is placed symmetrically on both sides of the feed line to shield the antenna from X-band system interference, which is step-4 (Figure 3d). Here, the bandwidth of the shielded band is widened by adjusting the U-shaped resonator parameters and etching the rectangular slots onto the ground plane.

**Figure 6:**
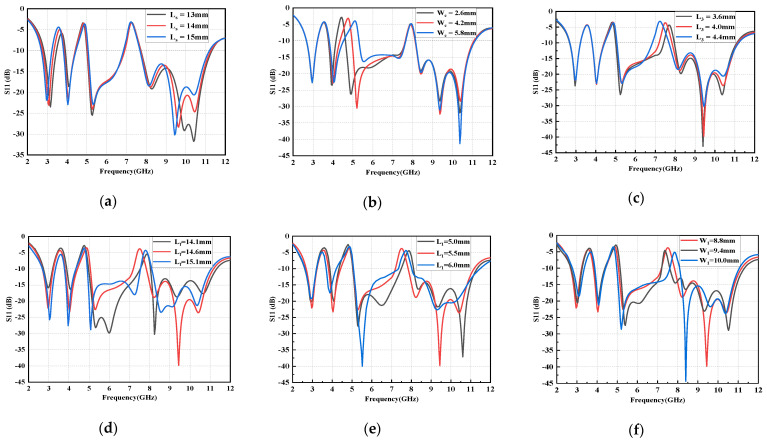
Tuning parametric study: (**a**) L_s_, (**b**) W_g_, (**c**) L_3_, (**d**) L_f_, (**e**) L_1_, (**f**) W_1_.

Next, the effects of different geometrical parameters of the proposed antenna on the band notch characteristics are studied, as portrayed in Figure 6. Evidently, with decreasing L_s_, the corresponding notched band becomes smaller, while the operational band remains unchanged. Similarly, by adjusting the size of inverted C-shaped slot opening, the entire second notch band can be shifted from a low frequency to a high frequency. Moreover, as the L_3_ increases, the range of the notch band becomes wider and the center frequency of the notch band shifts down. The dimension of L_f_ effects the phase of the antenna, while L_1_ and W_1_ determine the area of the radiation patch. When they increase or decrease, the resonant frequency shifts significantly in the range of 5.2−7.4 GHz and 8.1−11.0 GHz. Accordingly, it can be concluded that, with the proposed design approach, the notched frequency bands can be easily achieved and controlled to meet the practical requirements by merely adjusting the dimensions and locations of the resonating elements. Meanwhile, it is worthwhile noting that changing the parameters of resonators (notch elements) affects only the notch bands, and the return loss in the rest of the UWB frequency band remains virtually unchanged.

## 3. Results and Discussion

### 3.1. Fabrication and Measurement

**Figure 7:**
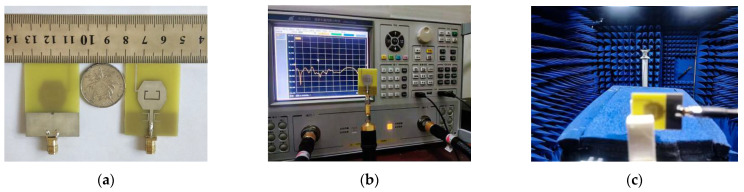
The proposed UWB antenna: (**a**) Fabricated prototype, (**b**) S11 measurement environment, (**c**) Pattern measurements in anechoic chamber.

To validate the proposed antenna design, an ultra-wideband antenna was successfully fabricated and tested according to the dimensions listed in Table 1. Figure 7a shows the prototype of the proposed antenna. Figure 7b presents the environment for S-parameter measurement. The measurement environment of the radiation pattern and peak gain is shown in Figure 7c.

**Figure 8:**
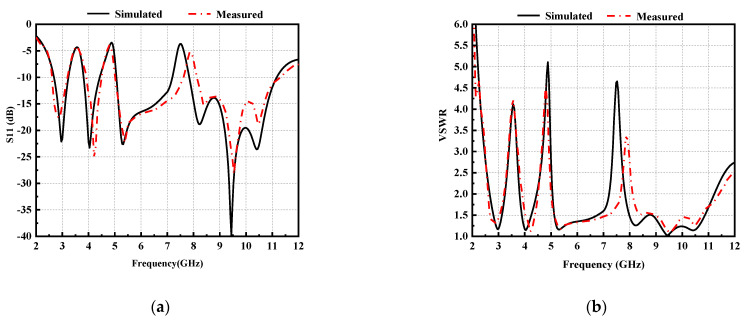
Simulated and measured results: (**a**) S11, (**b**) VSWR.

Figure 8 displays the measured and simulated results of S11 and VSWR. From the comparison of curves in Figure 8, it can be observed that the measurement results are well matched with the simulation results. The designed antenna covers the entire UWB frequency band for VSWR ≤ 2, except in the notched bands. At the center frequency of notch band, S11 > −5 dB and the value of VSWR > 3.3, thereby indicating the desired notch performance.

### 3.2. Radiation Characteristics

**Figure 9:**
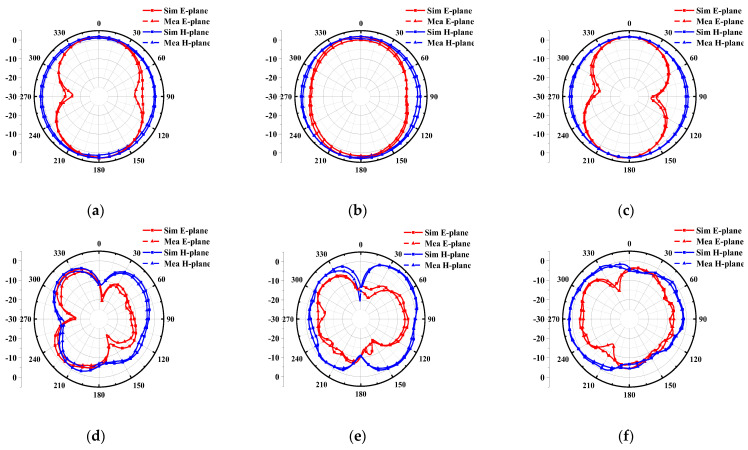
Simulated and measured radiation patterns at (**a**) 3 GHz, (**b**) 4 GHz, (**c**) 5.3 GHz, (**d**) 8.3 GHz, (**e**) 9.4 GHz and (**f**) 10.3 GHz.

Furthermore, radiation patterns are plotted in Figure 9. The proposed antenna possesses almost omnidirectional radiation on the H-plane (yoz-plane) and 8-shaped bidirectional radiation on the E-plane (xoz-plane), at low frequencies (3 GHz, 4 GHz and 5.3 GHz). However, with the increase in frequency, higher-order modes are generated that lead to an uneven phase distribution of the antenna. Therefore, the radiation patterns at 8.3 GHz, 9.4 GHz and 10.3 GHz become distorted.

**Figure 10:**
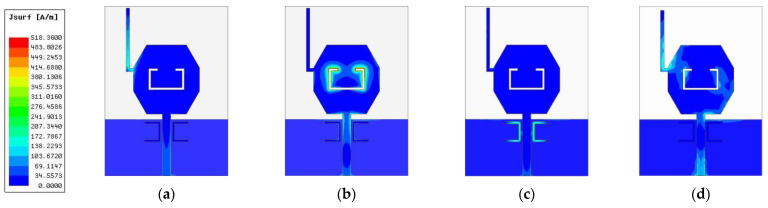
Current distribution in the antenna at (**a**) 3.5 GHz, (**b**) 4.9 GHz, (**c**) 7.9 GHz, (**d**) 4.0 GHz.

Figure 10 displays the surface current distribution of the proposed antenna. At 3.5 GHz, the current distribution is weak in the patch area, while it is strong at the L-shaped stub. Conversely, at 4.9 GHz, the current distribution is only strong at the feed line and the inverted C-shaped slot. Similarly, at 7.9 GHz, the currents are mainly distributed around the U-shaped patches and are oppositely directed between the interior and exterior edges. Therefore, the resultant radiation fields can be canceled out, and high attenuation near the resonant frequency is achieved, thus resulting in a notched band. Besides, the maximum surface current density is on the upper-left and lower-right corner of the octagonal patch at 4.0 GHz. Therefore, we can conclude that the notched structures exert significant effects on the current distribution of the antenna.

**Figure 11:**
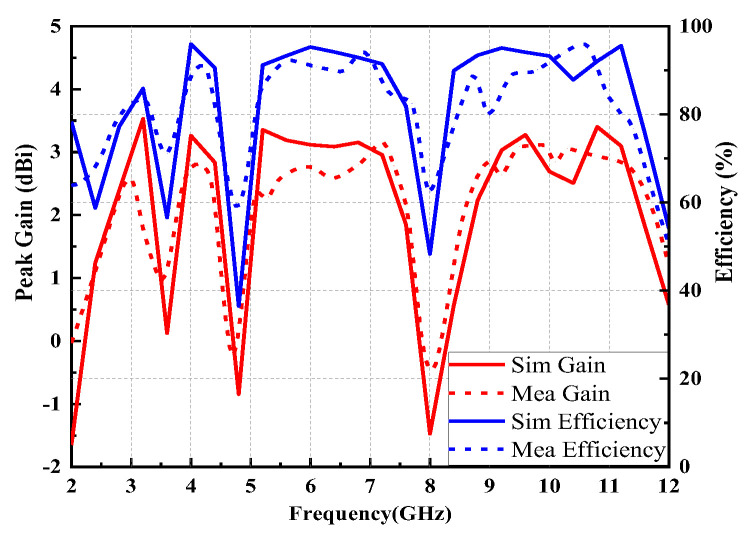
Peak gain and radiation efficiency of the proposed antenna.

Figure 11 progressively demonstrates the peak gain and radiation efficiency results of the proposed antenna. The efficiency of the proposed antenna is essentially higher than 80% over the UWB operating band, except for in the notched band, implying that the majority energy is radiated away. Meanwhile, the average in peak gain values is around 2.88 dBi in the passband. It is worth noting that both curves reduce drastically in the notched bands. At notch frequencies, impedance mismatch of the proposed antenna leads to the signal source energy not being fully absorbed and the formation of standing waves on the transmission line. As a result, the efficiency of the proposed antenna drops drastically. Concluding the foregoing discussions, the suggested UWB antenna has good radiation characteristics.

### 3.3. Comparison with Already Reported Works

Table 3 presents a comparison of the proposed antenna with other related literature in terms of dimensions, impedance bandwidth, notch technique, and applications. In contrast with the previously reported antennas, the proposed antenna achieves more notch bands where each notch band results from a different notch technique and has acceptable band-notched characteristics.

## 4. Conclusions

In this work, a compact planar monopole ultra-wideband antenna with anti-interference characteristics has been presented for UWB applications. Interestingly, the proposed antenna provides a wide impedance bandwidth, ranging from 2.70 GHz to 11.06 GHz. In addition, there was a discussion of three rejection bands around 3.22~3.83, 4.49~5.05 and 7.18~7.84 GHz for the applications of the WiMAX, INSAT and X-band. These were created by introducing an L-shaped stub in the radiation patch, a pair of U-shaped parasitic elements beside the feed line, and an etched inverted C-shaped slot. Additionally, the designed antenna had a simple structure and easy fabrication process. The antenna also possessed an acceptable peak gain and efficiency, demonstrating that the proposed antenna was certainly applicable in miniaturized devices for the the operation of UWB communication systems.

## Figures and Tables

**Table 1:** Optimized dimensions of the proposed antenna (unit: mm).

Parameter	Value (mm)	Parameter	Value (mm)	Parameter	Value (mm)	Parameter	Value (mm)
L	40	L_4_	8	L_s_	15	L_p_	5.4
W	29	L_f_	14.6	W_s_	0.8	W_p_	3.1
h	1.6	W_f_	2	G_l_	1	L_u_	0.5
L_1_	5.5	W_1_	9.4	G_w_	1.4	W_u_	3.8
L_2_	4	W_2_	8.4	L_c_	0.5	W_c_	4.2
L_3_	4.8	L_g_	13.3				

**Table 2:** The rejection bands and operational comparison for the evolution steps.

Step No.	No of Rejection Bands	Coverage of Rejection Bands (GHz)	No of Operational Bands	Coverage of Operational Bands (GHz)
1	0	-	1	3.29~12.04
2	1	3.14~3.86	2	2.70~3.14, 3.86~11.58
3	2	3.11~3.78, 4.66~5.10	3	2.62~3.11, 3.78~4.66, 5.10~11.68
4	3	3.22~3.83, 4.49~5.05, 7.18~7.84	4	2.70~3.22, 3.83~4.49, 5.05~7.18, 7.84~11.06

**Table 3:** Comparisons between the proposed triple-band-notched UWB antenna and other works.

Reference	Dimensions (mm)	Impedance Bandwidth (GHz)	Notch Band Application	Notch Technique	The Lowest Operating Frequency Ranges (GHz)
[27]	32 × 26 (0.30λ × 0.24λ)	2.8~11	WiMAX and WLAN	T-shaped stub and parasitic strips	2.8–3.3
[28]	29 × 40 (0.30λ × 0.41λ)	3.1–11	WLAN	Split-ring resonator	3.1–5.4
[29]	90.5 × 60.1 (0.93λ × 0.62λ)	3.1–10.6	WLAN	Absorptive bandstop filter	3.1–4.9
[30]	18 × 17 (0.17λ × 0.16λ)	2.9~12	INSAT and X-band	C slot, U slot	2.9–4.1
[31]	35 × 33 (0.32λ × 0.30λ)	2.7~10.6	C-band and WLAN	Modified V slot and EBG structure	2.7–3.5
[32]	18 × 36 (0.20λ × 0.26λ)	2.9~20	C-band	T-shaped stub	2.9–3.6
[33]	21 × 16 (0.26λ × 0.20λ)	3.77~11.64	WLAN and X-band	Symmetrical L-structured parasitics and S slot	3.77–5.6
[34]	32 × 14 (0.32λ × 0.14λ)	3~12	WLAN and X-band	Multimode resonator	3–5.2
[35]	30 × 35 (0.31λ × 0.36λ)	3.1~10.6	WLAN and X-band	Multimode resonator	3.1–4.0
Proposed work	40 × 29 (0.36λ × 0.26λ)	2.70~11.06	WiMAX, INSAT and X-band	C slot, resonator and parasitic stub	2.7–3.2

## Data Availability

Not applicable.
